# Understanding crop genetic diversity under modern plant breeding

**DOI:** 10.1007/s00122-015-2585-y

**Published:** 2015-08-06

**Authors:** Yong-Bi Fu

**Affiliations:** Plant Gene Resources of Canada, AAFC Saskatoon Research Centre, 107 Science Place, Saskatoon, SK S7N0X2 Canada

## Abstract

*****Key message***:**

**Maximizing crop yield while at the same time minimizing crop failure for sustainable agriculture requires a better understanding of the impacts of plant breeding on crop genetic diversity. This review identifies knowledge gaps and shows the need for more research into genetic diversity changes under plant breeding.**

**Abstract:**

Modern plant breeding has made a profound impact on food production and will continue to play a vital role in world food security. For sustainable agriculture, a compromise should be sought between maximizing crop yield under changing climate and minimizing crop failure under unfavorable conditions. Such a compromise requires better understanding of the impacts of plant breeding on crop genetic diversity. Efforts have been made over the last three decades to assess crop genetic diversity using molecular marker technologies. However, these assessments have revealed some temporal diversity patterns that are largely inconsistent with our perception that modern plant breeding reduces crop genetic diversity. An attempt was made in this review to explain such discrepancies by examining empirical assessments of crop genetic diversity and theoretical investigations of genetic diversity changes over time under artificial selection. It was found that many crop genetic diversity assessments were not designed to assess diversity impacts from specific plant breeding programs, while others were experimentally inadequate and contained technical biases from the sampling of cultivars and genomes. Little attention has been paid to theoretical investigations on crop genetic diversity changes from plant breeding. A computer simulation of five simplified breeding schemes showed the substantial effects of plant breeding on the retention of heterozygosity over generations. It is clear that more efforts are needed to investigate crop genetic diversity in space and time under plant breeding to achieve sustainable crop production.

**Electronic supplementary material:**

The online version of this article (doi:10.1007/s00122-015-2585-y) contains supplementary material, which is available to authorized users.

## Introduction

Plant breeding since the early 1900s has made a profound impact on food production and will continue to play a vital role in the world food security (Borlaug 1983; Tester and Langridge 2010). However, it has also introduced crop uniformity across the farm fields, which is genetically vulnerable to biotic and abiotic stresses (Day 1973; Duvick 1984; Vellve 1993; Tripp 1996; Keneni et al. 2012). Such risks have been well documented with the occurrence of epidemics such as the Irish potato blight in the 1840s and the U.S.A. corn blight in the 1970s (National Academy of Sciences 1972; Ullstrup 1972). The threat of the extremely virulent new race of stem rust Ug99 from East Africa to genetically uniform wheat is currently evident (Borlaug 2007; Babiker et al. 2015). Thus, it is important, although challenging, to compromise between maximizing crop yield under a given set of conditions and minimizing the risk of crop failure when conditions change and to develop effective strategies for sustainable agriculture (Hallauer 1985; Shukla and Mattoo 2013). Such a compromise requires a better understanding of the impacts of modern plant breeding on crop genetic diversity (Duvick et al. 2004; Fu 2006).

Efforts have been made over the last three decades to assess crop genetic diversity using molecular marker technologies. These assessments have generated considerable knowledge about the extent and nature of genetic diversity present in conserved and/or actively utilized germplasm of various crops (Rauf et al. 2010). These assessments not only facilitate our efforts in germplasm conservation, but also provide guidance for better germplasm utilization for genetic improvement. However, some assessments have also revealed some temporal patterns of crop genetic diversity (e.g., see van de Wouw et al. 2010a, b) that are largely inconsistent with our perception that modern plant breeding reduces crop genetic diversity (Fu et al. 2003; Gepts 2006). For example, a metaanalysis of 44 published diversity assessments indicated that a gradual narrowing of the genetic base of the varieties released by breeders could not be observed (van de Wouw et al. 2010b). One would expect that the intensive selection in modern plant breeding programs within a narrow range of plant germplasm with limited allele introgressions over time (Hallauer 1985; Allard 1999) would have reduced genetic diversity. Also it is evident that newly released crop varieties are phenotypically more uniform than before, implying a genetic diversity reduction (e.g., Duvick 1984; Bowman et al. 2003). Such a discrepancy suggests that we may still be far away from understanding genetic diversity of crops developed under modern plant breeding.

In this review, we attempt to explain this discrepancy by examining empirical assessments of crop genetic diversity and theoretical investigations on genetic diversity changes under artificial selection. Specifically, we hope to address the following questions: (1) Why diversity assessments have often not revealed diversity reduction from modern plant breeding? (2) Does plant breeding truly reduce crop genetic diversity? (3) How much is known theoretically about genetic diversity changes under artificial selection? and (4) What research is needed to fill the knowledge gap in this area of study? We have organized the review to address these questions with our arguments and thoughts.

## Empirical assessments of crop genetic diversity

Over the last three decades, concerns have been expressed about crop uniformity (Duvick 1984; Vellve 1993; Swanson 1996; Tripp 1996) and there have been an increased number of crop genetic diversity assessments (Donini et al. 2000; Reeves et al. 2004; Fu 2006; Rauf et al. 2010). Earlier assessments based on phenotypic (Rodgers et al. 1983; Ortiz et al. 2003) and pedigree data (Cox et al. 1985; van Beuningen and Busch 1997) had demonstrated that the substantial progress achieved in improving yield and other traits resulted in a reduction in the genetic diversity of improved gene pools (Cox et al. 1986; Smith et al. 2004). Advances in molecular markers such as random amplified polymorphic DNAs (RAPDs), amplified fragment length polymorphisms (AFLPs), and simple sequence repeats (SSRs) have made crop diversity assessments more attainable and informative than before. These molecular assessments, although rarely using genome-wide SNP markers, have generated a lot of knowledge about the nature and extent of genetic diversity present in various crops. Specific reviews on these assessments have been made with respect to marker application (Mondini et al. 2009) and crop genetic diversity (Reeves et al. 2004; Fu 2006; van de Wouw et al. 2010a; Rauf et al. 2010). The highlights of their findings are summarized in the following.

Fu (2006) reviewed 23 articles with the applications of RAPDs, AFLPs, and SSRs that were published from 2000 to 2005 in eight major journals associated with plant breeding. These articles revealed different impacts of plant breeding on improved gene pools, not only narrowing or widening their genetic base, but also resulting in genetic shifts. Overall, the genome-wide reduction of crop genetic diversity accompanying genetic improvement over time was minor, but allelic reduction at individual chromosomal segments was substantial. This review was not exhaustive, but focused more on the understanding of the impact of plant breeding on the genome.

van de Wouw et al. (2010a) reviewed about 110 publications associated with crop genetic diversity and agricultural modernization and concluded that different views exist on the concept of crop genetic erosion. Genetic erosion of cultivated diversity occurs in two stages: the initial replacement of landraces by modern cultivars; and further trends in diversity as a consequence of modern breeding practices. Genetic erosion may also occur at three levels of integration: crop, variety, and allele. They further argued that there is a reduction in diversity due to the replacement of landraces by modern cultivars, but no further reduction after this replacement has been completed. To support their argument, they performed a metaanalysis of 44 published papers and showed that a gradual narrowing of the genetic base of the varieties released by breeders was not observed (van de Wouw et al. 2010b). Specifically, a significant diversity reduction of 6 % occurred before the 1960s, and after the 1960s, increased diversity was found from plant breeding.

Rauf et al. (2010) reviewed about 230 publications associated with plant breeding and genetic diversity to understand the diversity impacts of different plant methods such as introduction, selection, and hybridization. In general, this review showed that losses of genetic diversity occurred but followed spatial and sometimes temporal trends. More losses of genetic diversity were found in released cultivars, followed by wild germplasm and landraces. Different plant breeding methods showed different impacts on plant genetic diversity. Plant introduction increased genetic diversity. Selection enhanced genetic differentiation at the expense of genetic diversity. Intraspecific hybridization lowered genetic diversity.

**Fig. 1:**
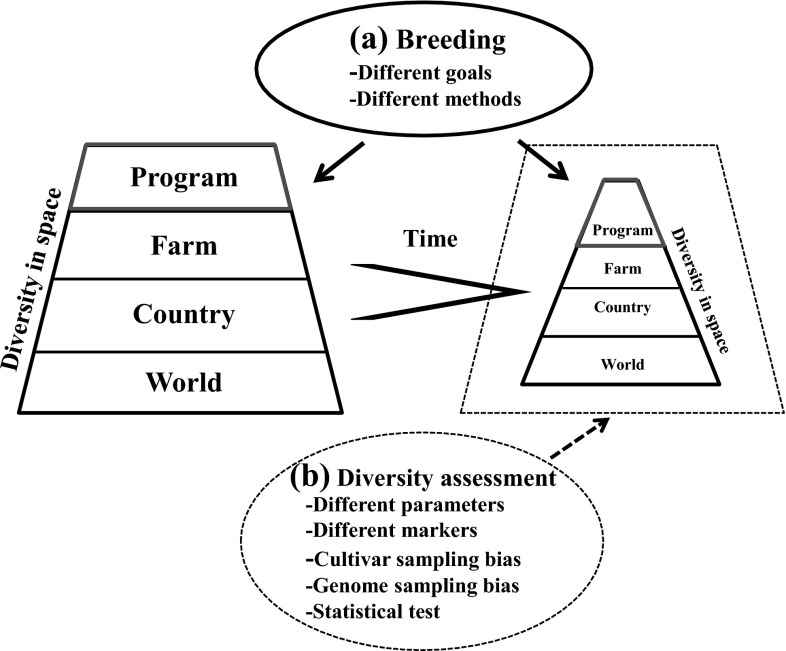
Illustration of the spatial and temporal changes (*solid line* and some highlight in *red*) in crop genetic diversity generated by modern plant breeding with variable goals and methods (**a**) and how they are obscured (*broken line*) under diversity assessments of variable nature (**b**) (color figure online)

It is clear that no consensus has been reached on the overall impact of modern plant breeding on crop genetic diversity. The temporal patterns of crop genetic diversity are largely inconsistent with our perception that modern plant breeding reduces crop genetic diversity (Gepts 2006) and are also incompatible with the fact that newly released crop varieties become phenotypically more uniform (Duvick 1984; Bowman et al. 2003). Such a discrepancy suggests that we may still be far away from understanding crop genetic diversity under modern plant breeding. An attempt was made here to explain this discrepancy as outlined in Fig. 1 and discussed below.

### Nature of modern plant breeding

Plant breeding since 1900s has had a profound impact on food production through developing and deploying new cultivars on a worldwide basis (Borlaug 1983). New cultivars have been developed through applications of many effective breeding methods, ranging from introduction, phenotypic selection on natural variants, selection with controlled mating, to marker-assisted selection for desirable genes (Allard 1999). The core of all plant breeding can be characterized as follows: (1) the conscious introduction of genetic diversity into breeding populations by intercrossing selected plants with outstanding characters that complement one another and; (2) the selection of superior plants with genes for desired traits until higher levels of improved adaptation, genetic uniformity, and agronomic stability are reached (Breseghello 2013). The choice for use of a breeding methodology is determined mainly by the mode of crop reproduction (selfing or crossing) and the breeding objectives to be achieved.

Crop breeding has been largely aimed at the improvement of yield, adaptation, resistance to biotic and abiotic stresses, and end-use quality. However, breeding objectives have changed over the years beyond yield improvement. New cultivars need to be developed with the capacity to achieve high yields in reduced chemical-input systems and with the genetic diversity needed to maintain yield stability under fluctuating climatic conditions (Heinemann et al. 2014). Many novel traits improved for sustainable agriculture include improved weed suppression ability, enhancement of nutritional value, and optimization of plant interactions with microbial communities in the soil, among others (e.g., see Brummer et al. 2011). To meet these challenges, conventional plant breeding has evolved by adopting approaches from different scientific disciplines, allowing breeders to increase their efficiency and exploit genetic resources more thoroughly. Among these new approaches are haploid generation (Kasha and Kao 1970); the use of sterility systems and transgenic technology (Salick 1995); apomixis (Spillane et al. 2004); and molecular marker-assisted breeding (Moose and Mumm 2008).

Modern plant breeding has been evolving from conventional breeding to molecular breeding for various breeding goals and diverse breeding methods have been applied over time (Gepts and Hancock 2006). As a consequence, selective pressure within breeding populations differs at various breeding stages for different breeding programs, so genetic diversity present in released cultivars of a crop may vary (Rauf et al. 2010). More heterogeneity is expected in varietal genetic diversity between selfing and outcrossing crops.

### Variation in genetic diversity measures

To our knowledge, genetic diversity is a term not well defined. It is broadly referred to as any variation in the nucleotides, genes, chromosomes, or genomes of a species at a level of individual, population, species, or region for a given time. Such a broad definition certainly invites misunderstanding and misinterpretation. Accordingly, its measurements are not unique (Mondini et al. 2009). Genetic diversity within a population is commonly measured by (1) allelic polymorphism; (2) heterozygosity; and (3) theta parameter (*θ* = 4*Neμ* for diploid genes, where *Ne* is effective population size and *μ* is per generation mutation rate). Genetic variation among populations, reflected in the differences in allele and genotype frequencies, is frequently measured using several different metrics. They are (1) *F*st and analogs; (2) genetic distance such as Nei’s D; and (3) sequence divergence (e.g., see Hedrick 2011). There has been considerable discussion on the proper uses of these diversity measures (e.g., see Jost 2008; Whitlock 2011).

Crop genetic diversity has traditionally been analyzed using morphological traits, particularly those agro-morphological traits of interest to users. To minimize the impact of environmental factors in the analysis, biochemical techniques such as isozyme and protein electrophoresis (Hunter and Merkert 1957) were later employed. Since 1990, various molecular techniques such as RAPD, AFLP, and SSR, have been used to measure genetic variation (Mondini et al. 2009). These molecular markers not only avoid the influence of environment, but also provide better sampling of the plant genome, thus increasing the resolution of measurements of genetic variation. Currently, there are more than 30 types of molecular markers available for assessing genetic diversity (Mondini et al. 2009). These markers have been widely applied to measure genetic diversity in crop plants and have played an important role in the characterization of crop genetic variation. However, genome-wide SNP markers with better sampling of plant genomes have not fully been applied to assess crop genetic diversity (Hyten et al. 2006).

Based on the broad definition of genetic diversity, the use of different diversity parameters, and the application of different molecular markers, it is not surprising that there is considerable heterogeneity in reported diversity assessments (Fu 2006; Aremu 2011). It is difficult to interpret and generalize the findings from estimation of different diversity parameters using different markers, even on a crop species (Rauf et al. 2010). Specifically, not all of the genetic diversity measures applied have been equally sensitive in detecting diversity changes from plant breeding practices, and different diversity measures may have different levels of accuracy and precision (Mohammadi and Prasanna 2003; Fu et al. 2005). Not all of the molecular markers applied have been equally informative for diversity assessments, as illustrated in oat using AFLP and SSR markers (Fu et al. 2003, 2004). Thus, discrepancies can be expected, even for the same assessment using different diversity parameters.

### Technical considerations in diversity assessment

An analysis of the published diversity assessments since 2000 shows that a majority of published assessments were not aimed specifically at the assessment of the diversity impacts of individual breeding programs or schemes on released cultivars. For example, many assessments were made to evaluate spatial and temporal patterns of genetic diversity present among cultivars released from different breeding programs, regions, or countries (e.g., see Roussel et al. 2005; Orabi et al. 2014). These assessments can inform us in general about the nature and extent of existing crop genetic diversity for exploration of conservation strategies and for germplasm utilization in plant breeding, but cannot provide us with much evidence that modern plant breeding reduces crop genetic diversity. The informative assessments would be those that evaluate the genetic diversity changes in all the released cultivars over all the breeding periods from individual breeding programs (e.g., see Fu et al. 2003). Alternatively, the diversity comparison between on-farm landraces present before a breeding program and on-farm cultivars released over time from the breeding program would also generate findings useful for evidence of genetic diversity reduction (e.g., see Russell et al. 2000). However, there are not many such assessments available to make firm inferences due to many practical and/or unknown reasons as shown in Fig. 1 and discussed below (also see Fu 2006; Rauf et al. 2010).

Many reported temporal diversity analyses are technically not ideal to address the diversity impacts of plant breeding on released cultivars. Technical biases could further mask the reported patterns of crop genetic diversity. Several issues have been identified (Fu 2006; Aremu 2011) and they include bias of sampling cultivars from a specific breeding program, arbitrary grouping of cultivars to represent specific breeding periods, mingling of gene pools from different breeding programs, applications of different markers that are variably informative, use of different genetic diversity measures, and inadequate statistical tests of significance. For example, bias may occur in sampling cultivars. Some older, important cultivars may have been lost; some newly developed cultivars may not be accessible; and selection may favor dominant, but genetically related, cultivars (Koebner et al. 2003; Le Clerc et al. 2005). Another example is the arbitrary grouping of assayed cultivars to represent specific breeding periods or inadequate separation of the change from landraces to cultivars from modern plant breeding (Reif et al. 2005).

The lack of statistical tests for significance in many reported assessments, even with useful test methods available (e.g., see Fu et al. 2003; Fu 2010), is another notable issue, adding little confidence to the interpretation of the diversity changes, and complicating generalization of published findings. Allelic counts for various groups are sensitive to the cultivar number for each group (Fu 2010), but some SSR studies did not correct the bias of unbalanced group sizes (e.g., see Russell et al. 2000; Duvick et al. 2004), thus weakening the argument for the allelic reductions found (Lu and Bernardo 2001; Fu et al. 2003).

### Informative assessments of diversity changes

Fortunately, informative assessments of diversity changes are available. Here we highlight a few cases to illustrate the importance of assessing specific, large-scale, long-term breeding programs for understanding diversity reduction from plant breeding.

A notable and commendable effort is the assessment of the diversity changes under recurrent selection (RS) schemes over many generations (Brown and Allard 1971), as RS has been widely used for maize improvement, while simultaneously maintaining genetic variability for continued selection since 1939 (Hallauer and Miranda 1988). Several investigations using molecular markers have yielded a clear picture on the diversity impacts of RS practiced over many generations (e.g., see Labate et al. 1999; Pinto et al. 2003; Hinze et al. 2005; Solomon et al. 2010; Romay et al. 2012). For example, Labate et al. (1999) investigated temporal changes in RFLP alleles over 12 generations in two reciprocally selected maize populations and reported about 10 % of the original RFLP alleles fixed and about 40 % of the total heterozygosity lost between generations 0 and 12. Similarly, Solomon et al. (2010) assessed SSR variation over 11 generations of RS in tropical maize breeding populations and found 33 % alleles lost and an 18 % reduction of within-population variance from generation 0.

Duvick et al. (2004) made a considerable effort to understand the diversity changes in the long-term commercial plant breeding program of Pioneer Hi-Bred International by using 969 SSR alleles to quantify the diversity changes in the Era hybrids and open-pollinated cultivars that were sequentially released in the west-central U.S. Corn Belt from the 1930s to 2000s. They found that the number of alleles fluctuated from decade to decade; that about 40–50 % of the 969 alleles were present in any one decade; and that there is a weak trend toward lower numbers per decade, starting in the 1980s. Based on the SSR polymorphism data, they also revealed a trend toward reduction in the average number of alleles per locus and a clear divergence between the allele profiles of the inbreds created by pedigree breeding in the Stiff Stalk and Non-Stiff Stalk heterotic groups.

The Illinois long-term selection experiment for grain protein and oil concentrations in maize has undergone 114 generations of recurrent selection since 1896, making it the longest running continuous genetics experiment in higher plants (Moose et al. 2004). Mikkilineni and Rocheford (2004) conducted a RFLP analysis of 200 plants selected from generations 65 and 91 of the Illinois High and Low Protein (IHP and ILP, respectively) strains and from generations 69 and 91 of the Reverse High and Low Protein (RHP and RLP, respectively) strains. Based on 35 RFLP probes, they found the percentage of RFLP probes with a variant fixed ranged from 22.9 to 51.4 % over 26 generations of selection for IHP; 25.7–42 % for ILP, and from 14.3 to 17.1 % over 22 generations of selection for RHP, and 14.3–22.9 % for RLP. Measuring the loss of heterozygosity relative to the original heterozygosity in generations 65 or 69 revealed that the IHP strain at generation 91 had lost 36 % of the original heterozygosity, followed by ILP 23 %, RLP 10 %, and RHP 3 %.

We conducted a series of genetic diversity analyses from 1999 to 2009 focusing on the Canadian gene pools of flax, oat, wheat, soybean, potato, and canola that were established over the last century and summarized these case studies in a book chapter of genetic erosion and biodiversity (Fu and Dong 2015). The Canadian crop gene pools displayed variable patterns and degrees of genetic diversity decline over the past 100 years breeding efforts. For example, we performed a SSR-based diversity analysis of 75 Canadian hard red spring wheat cultivars released from 1845 to 2004 by several Canadian wheat breeding programs with similar breeding goals and methods (Fu and Somers 2009). We found that (1) significant allelic reduction started as early as the 1930s; (2) 38 % of 2010 SSR alleles detected in the 20 cultivars released before 1910 were retained, 18 % are new, and 44 % were lost in the 20 cultivars released after 1990; (3) allelic reduction occurred in every part of the wheat genome and a majority of the reduced alleles resided in only a few early cultivars; (4) a significant genetic shift was also observed in the gene pool in response to the long-term breeding pressure; and (5) these allelic changes were associated with long-term wheat trait improvements (Fu and Somers 2011).

## Theoretical investigations on diversity changes under artificial selection

Modern breeding programs of plants and animals have heavily relied on the theories of quantitative genetics and artificial selection in finite populations as guides to improve quantitative traits of interest (Falconer and Mackay 1996; Hill 2010). These theories allow us to understand the genetic basis of a quantitative trait, the prediction of selection responses, the limit of artificial selection, and the maintenance of genetic variation at the selected loci (Lynch and Walsh 1998). Discussing all of these theories is beyond the scope of this review, except the relevant and influencing theory of selection limit (Robertson 1960). This theory states that artificial selection in a small population, such as a plant breeding population, is expected to increase the frequency of favorable alleles, along with the chance fixation of other less desirable and selectively neutral alleles. Consequently, considerable efforts have been made to investigate the effects of selection and genetic drift in finite populations from theoretical reasoning, computer simulation, and empirical evaluation (Jones et al. 1968; Hill and Caballero 1992; Wray and Goddard 1994; Walsh 2004; Hill 2014).

However, our literature search revealed several interesting observations. First, previous research into selection and drift in finite populations focused more on selection response and limit, and less on companying diversity change (Hill 2014), so diversity dynamics in small populations are poorly understood. Second, some theoretical queries investigated diversity changes at selected or relevant loci through genetic variance, and did not consider the diversity impacts on genetic background through linkage and recombination (Hill and Robertson 1966; Felsenstein 1974; Charlesworth et al. 1993), so a little is known about the genome-wide diversity changes under artificial selection. Third, little attention has been paid to theoretical queries on the long-term diversity impact from specific breeding methods (Fu et al. 1998), as plant breeding programs often employ a mix of different artificial selection procedures over breeding periods to reach breeding goals.

### Heterozygosity changes in a small population

**Fig. 2:**
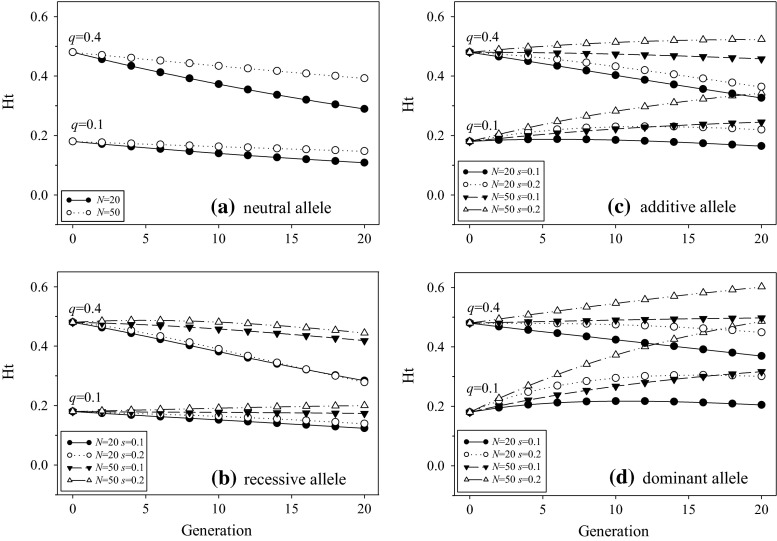
Predicted heterozygosity (*H*_t_) over generations for an allele of various characteristics (**a** neutral alleles; **b** recessive allele; **c** additive allele; and **d** dominant allele) under genetic drift and/or selection in a finite population of size *N*. The prediction for neutral allele (**a**) was obtained from the Eq. (1) and the predictions for **b**–**d** from the Eq. (2). The predictions in **b**–**d** are specified at a selective locus with two allele frequencies (*q* = 0.1, 0.4), two population sizes (*N* = 20, 50), two selection coefficients (*s* = 0.1, 0.2), and three levels of dominance [*h* = 0 (recessive), 0.5 (additive), and 1 (dominant)]

If modern plant breeding can be characterized as the directional selection that humans have performed in a small population to improve a trait of interest, the early theoretical research on small populations, particularly for those by Wright-Fisher, is highly relevant. Related population genetic theories for small populations (Hedrick 2011) predict that small population size can lead to the loss of neutral genetic variation and fixation of mildly deleterious alleles, thereby reducing population fitness. The predicted heterozygosity (*H*_t_) due to genetic drift alone is

1 $$H_{\text{t}} = 2q_{0} (1 - q_{0} )\left( {1 - \frac{1}{2N}} \right)^{t} ,$$

where *q*_0_ is the initial allele frequency, *N* is the population size, and *t* is the generation (Hedrick 2011). A numerical illustration is given in Fig. 2a, where the heterozygosity for a neutral allele of frequency (*q* = 0.1 or 0.4) is predicted to reduce over generations in a population of size (*N* = 20 or 50). These predictions were empirically confirmed with some *Drosophila* experiments (e.g., see Buri 1956).

The single-locus theory of selection in a finite population was formulated by Kimura in 1957 using a diffusion model to predict the fixation of a favorable allele and was extended by Robertson (1960) to predict the limit (or selection potential) to directional selection. The effects of genetic drift and selection have been theoretically investigated on the selection responses and limits, but less on the maintenance of genetic variance (Arunachalam 1974). Silvela (1980) applied conditional probabilities and moment-generating matrices to derive the expected single-locus heterozygosity (*H*_t_) over generations of artificial selection with a finite population (see the Eq. (5) of Silvela 1980). Replacing with the directional selection fitness model (1 + *s*, 1 + *sh*, 1 for AA, Aa, aa genotypes), we can formulate his derived heterozygosity as below:

2 $$H_{\text{t}} = 2q_{0} (1 - q_{0} )A^{t} \left\{ {1 + \left[ {t + \frac{4N - 3}{5N - 3}\left( {\frac{{1 - B^{t} C^{t} }}{1 - BC} - t} \right) - \frac{8N - 6}{2N - 1}\frac{{1 - B^{t} C^{t} }}{1 - BC}q_{0} (1 - q_{0} )} \right]s\left( {h - \frac{1}{2}} \right) + \frac{{1 - B^{t} }}{1 - B}(1 - 2q_{0} )\frac{s}{2}} \right\},$$

where $A = (1 - \frac{1}{2N})$, $B = (1 - \frac{2}{2N})$, $C = (1 - \frac{3}{2N})$, *s* is the selection coefficient, and *h* is the level of dominance.

To understand the changes of expected heterozygosity over generations of directional selection in a finite population, Eq. (2) was specified with some sample parameter values and illustrated in Fig. 2b-d. It is clear that the heterozygosity for a favorable allele of moderate frequency (*q* = 0.4) in a smaller population (*N* = 20) will be reduced over generations, but could be increased in a larger population (*N* = 50). However, the expected heterozygosity for a favorable allele of low frequency (*q* = 0.1) in a small population can be maintained or increased over generations, depending on the type of alleles (or the level of dominance *h*). Both the population size and selection coefficient work in the same direction to enhance the heterozygosity changes (either decrease or increase) over generations. Generally, heterozygosity for a neutral allele in a small population will be reduced over generations, but those for a selective allele can be increased or decreased, depending on the initial frequency and type of alleles.

However, a little is known about the heterozygosity changes in a multi-locus system with linkage, epistasis, and inbreeding (Robertson 1970; Arunachalam 1974). Heterozygosity in a multi-locus system is known to be less sensitive to rare alleles and thus is a less accurate measure of genetic diversity (Crow and Kimura 1970). There are no direct theoretical models available to predict the allelic richness in a finite population under directional selection over generations (Caballero and García-Dorado 2013). Some theoretical studies, mainly using computer simulation (e.g., Hedrick 1970), have been conducted to predict the maintenance of genetic variation in quantitative traits under artificial selection (e.g., Hill and Rasbash 1986; Zhang et al. 2002), but we are still far from understanding the dynamics of genetic variance under artificial selection (Brotherstone and Goddard 2005; Hill 2014).

### Heterozygosity changes in a simulated breeding population

A breeding population may be more complex than the single-locus or multiple-loci model under directional selection in a small population as discussed above, because selection, genetic drift, non-random mating, migration, and mutation can all contribute and interact to alteration of genetic diversity (Falconer and Mackay 1996). Although studies on recurrent selection in maize breeding populations have advanced our understanding of diversity changes as mentioned above, insufficient attention has been paid to theoretical investigations of the diversity impact of specific breeding schemes. Much less is known on how a breeding scheme affects the genetic background, along with the selected loci (Allard 1988).

**Fig. 3:**
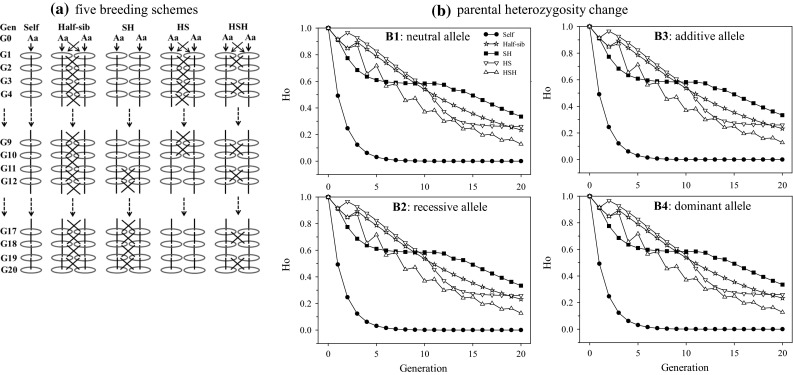
Breeding schemes (**a**) and parental heterozygosity (*H*_o_) changes (**b**) over 20 generations in simulated breeding programs to improve a quantitative trait of interest. The simulation considered five breeding schemes (Self = selfing; Half-sib = half-sib; SH = selfing + half-sib; HS = half-sib + selfing; HSH = half-sib + selfing + half-sib) and generated 50 diploid progeny in each generation with 5000 loci. The first 20 loci control the trait with four genetic models considered [neutral (*s* = 0, *h* = 0), recessive (*s* = 0.2, *h* = 0), additive (*s* = 0.2, *h* = 0.5), and dominant (*s* = 0.2, *h* = 1)]. The progeny with the largest genetic values were selected as parents for crossing and the parental heterozygotes were counted over 5000 loci. Environmental variation and its interactions with genotypes were not considered

To confirm our theoretical expectation on diversity reduction under plant breeding for this review, a computer simulation of a small breeding population developed for the improvement of a quantitative trait of interest was carried out and diversity changes over generations were assessed. In the simulation, five breeding schemes were applied to a breeding population over 20 generations (Fig. 3a). The breeding schemes, Self and Half-sib, used a pure mating type over generations (i.e., continuous selfing and half-sib mating, respectively) and the others (Self + Half-sib, Half-sib + Self, and Half-sib + Self + Half-sib) applied a mix of both selfing and half-sib mating, alternating over generations. These simplified breeding schemes, although knowingly deviating from existing plant breeding programs, were arbitrarily chosen mainly for theoretical confirmation (Fu et al. 1998).

We considered each individual had *n* unlinked selective loci each with two alleles a and A. At each locus, we assigned three genotypes aa, Aa, and AA to relative genetic values of 1, 1 + *hs*, and 1 + *s*, respectively, where *s* is the selective disadvantage of AA and *h* is the level of dominance. The genetic value of a progeny *i* with the trait was given in the multiplicative fitness model by the expression:

3 $$w_{i} = (1 + hs )^{y} (1 + s )^{z} ,$$

where *y* and *z* are the numbers of loci with Aa and AA in the progeny, respectively. We also assumed that selection took place in the diploid stage of the life cycle, that no mutation at these loci occurred during the period of breeding, and that selection and dominance parameters were the same at all loci. Moreover, environmental variation and its interactions with genotypes were not considered.

For the breeding scheme Self, the simulation started with a parental generation of two unrelated individuals for each replicate. For this pair of parents, one of the four initial alleles at each locus was randomly designated as having a selective disadvantage as a homozygote; the other three alleles had no disadvantage and were selectively equivalent. From each mating, progeny of given size were generated. For each progeny, the genotype was determined, locus by locus, by randomly choosing one of two gametes (with equal probability) from each of two parents to form a zygote. For each genotype, the numbers of homozygotes and heterozygotes of alleles were counted over loci, from which the relative genetic value of a progeny for the trait of interest was determined using Eq. (3), in combination with various sets of genetic parameter values. The progeny with the largest genetic value for each full-sib family was selected as the parent for the next generation and was self-fertilized to produce the G1 progeny. This process was followed for 20 generations and 100 replicates were run for each combination of genetic parameters and progeny sizes. In this study, 5000 loci were simulated and only the first 20 loci contributed multiplicatively to the trait of interest. Seven genetic models were examined: neutral (*s* = 0, *h* = 0), weakly recessive (*s* = 0.1, *h* = 0), strongly recessive (*s* = 0.2, *h* = 0), weakly additive (*s* = 0.1, *h* = 0.5), strongly additive (*s* = 0.2, *h* = 0.5), weakly dominant (*s* = 0.1, *h* = 1), and strongly dominant (*s* = 0.2, *h* = 1). Two progeny sizes (20, 50) were used. Thus, there were 14 combinations for each breeding scheme.

For the other breeding schemes, the same procedures as in Self were applied, but they differed in the use of two parental individuals heterozygous for all the loci to form two full-sib families and the selection of the two progeny with the largest genetic values for the trait as the parents of the next generation from each full-sib family, as described in the breeding schemes above. The simulations were done with an R script (R Development Core Team 2014) that was written specifically for this investigation and is available from the author upon request.

The simulated results on progeny size 50 are shown in Table S1 for parental genetic value improvements and Table S2 for heterozygosity changes. Several diversity patterns are clear. First, as expected, artificial selection improved the trait with increased genetic value (Table S1), while decreased the genetic diversity with reduced heterozygosity (Table S2). For example, the parental heterozygosity approached zero after seven successive selfings (Fig. 3b; Table S2). Second, comparisons among the five breeding schemes showed half-sib mating was the most effective scheme to achieve higher genetic gain with lower reduction of heterozygosity (Fig. 3b). Third, the genetic fitness models examined were associated with the trait improvement, but not directly with the patterns of heterozygosity reduction (Fig. 3b). Fourth, the patterns of trait improvement and diversity reduction were essentially the same for both progeny sizes of 20 and 50 (Fig. 3b). These findings help to confirm our expectation that plant breeding can theoretically reduce genetic diversity. Also, our simulation, although preliminary, appears to have utility in understanding gain potential and diversity dynamics under a breeding scheme.

## Future investigations into genetic diversity dynamics

Concerns about crop uniformity and the possible risk of crop failure (Ullstrup 1972; Day 1973) have continued in the past decade, particularly with the emerging threat of wheat stem rust Ug99 from East Africa (Borlaug 2007; Babiker et al. 2015). More questions have been raised concerning sustainability and innovation in staple crop production based on the narrowing genetic base (Kahane et al. 2013; Heinemann et al. 2014). Sustainable agriculture requires a compromise between maximizing crop yield and minimizing the risk of crop failure; thus, there is a need for a better understanding of the plant breeding impacts on crop genetic diversity. Here we propose several lines of research that would fill the gaps present in our knowledge of crop genetic diversity occurring under plant breeding.

First, a standardization of crop genetic diversity assessments should be sought to avoid technical biases identified in this review and applied for more informative assessment of genetic diversity dynamics (FAO 2013). It is technically feasible to develop and establish standard assessments, given the recent advances in next generation sequencing. Genotyping-by-sequencing methods are available to allow the acquisition of thousands of genome-wide SNPs in non-model plant species for diversity assessment (e.g., see Peterson et al. 2014). Heterozygosity should be measured and reported on SNP data. Also, computer simulations can be performed using different diversity parameters, genome coverages, and sampling strategies to evaluate the accuracy and precision of a diversity assessment.

Second, continuous assessments on crop genetic diversity using advanced genomic techniques are needed to facilitate the effective monitoring of newly released cultivars and on-farm crop diversity. This could be done with an assay of cultivars released over time from specific breeding programs and quantification of diversity changes. More emphasis should be given to the assessments of crop genetic diversity at the breeding program and farm levels, and less on country or world scale. More can be learned from the temporal diversity analysis of long-term public and commercial breeding programs such as the Illinois long-term maize selection experiment and Pioneer Hi-Bred International maize improvement program. Empirical assessments of genomic response to artificial selection like those in animal breeding (Flori et al. 2009; Kim et al. 2013) would provide more insight into breeding impacts on the plant genome. The major challenge is to determine whether the alleles eliminated from plant breeding are of any adaptive value, are genetically associated with any traits of future importance, or are linked to any selected genes at nearby loci (Fu and Somers 2009).

Third, theoretical research into trait improvement and diversity change under different breeding schemes should be pursued, particularly by computer simulation (Caballero and García-Dorado 2013). The preliminary simulation described in this review can be expanded to consider complex breeding schemes that are compatible with existing breeding programs. Genotype-by-environment interaction can be modeled to reflect the complex nature of a quantitative trait expressed in target environments (Marigorta and Gibson 2014). Focus should be placed on the development of breeding strategies that allow for continuous development of superior genotypes with minimal loss of genetic variation (Hallauer and Miranda 1988). These studies will not only enhance our understanding of crop genetic diversity dynamics under artificial selection, but also may generate guidance for plant breeders on how to compromise between maximizing crop yield and minimizing the risk of crop failure.

## Concluding remarks

Our review has highlighted the fact that crop genetic diversity under modern plant breeding has been improperly and insufficiently investigated, either from an empirical aspect or theoretical background, and thus is poorly understood. Many reported assessments of crop genetic diversity were not aimed at the assessment of diversity impacts from specific breeding programs and some were experimentally inadequate and had technical biases from the sampling of cultivars and genomes. Little attention has been paid to theoretical investigations on crop genetic diversity changes under plant breeding. A computer simulation of five simplified breeding schemes showed substantial breeding effects on the retention of heterozygosity over generations. It is clear that more efforts are needed to investigate crop genetic diversity in space and time under plant breeding for sustainable crop production.

### **Author contribution statement**

YBF conceived of the research, performed the computer simulation and wrote the paper.

## Electronic supplementary material

Supplementary material 1 (PDF 17 kb)
