# Supplementary material for: Understanding crop genetic diversity under modern plant breeding
Source: Theor Appl Genet. 2015 Aug 6;128(11):2131–42. doi: 10.1007/s00122-015-2585-y (PMC4624815; doi:10.1007/s00122-015-2585-y)
Supplement: Supplementary file 1 — Supplementary material 1 (PDF 17 kb) [file 122_2015_2585_MOESM1_ESM.pdf]

Yong-Bi Fu (2015) Understanding crop genetic diversity under modern plant breeding. Theoretical and Applied Genetics. **Supplemental online materials**

Table S1 Averaged parental genetic values achieved for the quantitative trait over 20 generations in simulated breeding programs with five breeding schemes under seven genetic models

Gen	0	1	2	3	4	5	6	7	8	9	10	11	12	13	14	15	16	17	18	19	20
Sn:	1	NRep:	100	Nloci:	5000	Sloci:	20	Ngen:	20	Nprgy:	50	Sel:	0	Dom:	0						
S	1.0	1.0	1.0	1.0	1.0	1.0	1.0	1.0	1.0	1.0	1.0	1.0	1.0	1.0	1.0	1.0	1.0	1.0	1.0	1.0	1.0
H	1.0	1.0	1.0	1.0	1.0	1.0	1.0	1.0	1.0	1.0	1.0	1.0	1.0	1.0	1.0	1.0	1.0	1.0	1.0	1.0	1.0
SH	1.0	1.0	1.0	1.0	1.0	1.0	1.0	1.0	1.0	1.0	1.0	1.0	1.0	1.0	1.0	1.0	1.0	1.0	1.0	1.0	1.0
HS	1.0	1.0	1.0	1.0	1.0	1.0	1.0	1.0	1.0	1.0	1.0	1.0	1.0	1.0	1.0	1.0	1.0	1.0	1.0	1.0	1.0
HSH	1.0	1.0	1.0	1.0	1.0	1.0	1.0	1.0	1.0	1.0	1.0	1.0	1.0	1.0	1.0	1.0	1.0	1.0	1.0	1.0	1.0
Sn:	2	NRep:	100	Nloci:	5000	Sloci:	20	Ngen:	20	Nprgy:	50	Sel:	0.1	Dom:	0						
S	1.7	2.8	3.5	3.7	3.7	3.7	3.7	3.7	3.7	3.7	3.7	3.7	3.7	3.7	3.7	3.7	3.7	3.7	3.7	3.7	3.7
H	1.6	2.0	2.4	3.4	4.4	5.2	5.9	6.2	6.3	6.4	6.4	6.4	6.4	6.4	6.4	6.4	6.4	6.4	6.4	6.4	6.4
SH	1.6	2.8	3.5	3.7	3.7	3.7	3.7	3.7	3.7	3.7	3.7	2.3	3.8	4.4	5.0	5.6	5.7	5.8	5.8	5.8	5.8
HS	1.7	2.0	2.4	3.5	4.4	5.3	5.9	6.2	6.4	6.4	6.5	6.5	6.5	6.5	6.5	6.5	6.5	6.5	6.5	6.5	6.5
HSH	1.6	2.0	2.4	4.0	4.0	5.4	5.4	6.2	6.2	6.5	6.5	6.5	6.5	6.5	6.5	6.5	6.5	6.5	6.5	6.5	6.5
Sn:	3	NRep:	100	Nloci:	5000	Sloci:	20	Ngen:	20	Nprgy:	50	Sel:	0.2	Dom:	0						
S	2.6	6.9	11.1	12.1	12.1	12.1	12.1	12.1	12.1	12.1	12.1	12.1	12.1	12.1	12.1	12.1	12.1	12.1	12.1	12.1	12.1
H	2.6	3.9	5.6	10.7	17.4	25.1	30.3	33.0	34.0	34.7	35.0	35.2	35.2	35.3	35.3	35.3	35.3	35.3	35.3	35.3	35.3
SH	2.6	7.0	10.9	11.8	11.8	11.8	11.8	11.8	11.8	11.8	11.8	4.9	13.0	17.2	21.6	27.2	28.6	29.3	29.4	29.6	29.7
HS	2.7	3.8	5.5	11.0	17.6	24.6	30.9	33.4	34.2	35.1	35.6	36.0	36.0	36.0	36.0	36.0	36.0	36.0	36.0	36.0	36.0
HSH	2.7	3.8	5.7	14.9	14.4	25.8	26.0	33.5	33.6	35.8	35.7	36.0	35.9	36.0	36.0	36.0	36.0	36.0	36.0	36.0	36.0
Sn:	4	NRep:	100	Nloci:	5000	Sloci:	20	Ngen:	20	Nprgy:	50	Sel:	0.1	Dom:	0.5						
S	2.7	3.2	3.8	4.0	4.0	4.0	4.0	4.0	4.0	4.0	4.0	4.0	4.0	4.0	4.0	4.0	4.0	4.0	4.0	4.0	4.0
H	2.6	3.3	4.2	5.1	6.0	6.6	6.7	6.7	6.7	6.7	6.7	6.7	6.7	6.7	6.7	6.7	6.7	6.7	6.7	6.7	6.7
SH	2.7	3.3	3.8	4.1	4.1	4.1	4.1	4.1	4.1	4.1	4.1	4.1	5.0	5.7	6.2	6.2	6.2	6.2	6.2	6.2	6.2
HS	2.7	3.3	4.2	5.1	5.9	6.6	6.7	6.7	6.7	6.7	6.7	6.7	6.7	6.7	6.7	6.7	6.7	6.7	6.7	6.7	6.7
HSH	2.6	3.2	4.2	5.2	5.9	6.6	6.7	6.7	6.7	6.7	6.7	6.7	6.7	6.7	6.7	6.7	6.7	6.7	6.7	6.7	6.7
Sn:	5	NRep:	100	Nloci:	5000	Sloci:	20	Ngen:	20	Nprgy:	50	Sel:	0.2	Dom:	0.5						
S	6.8	10.2	13.4	15.0	15.1	15.1	15.1	15.1	15.1	15.1	15.1	15.1	15.1	15.1	15.1	15.1	15.1	15.1	15.1	15.1	15.1
H	6.8	10.2	15.6	23.0	30.3	36.6	38.3	38.3	38.3	38.3	38.3	38.3	38.3	38.3	38.3	38.3	38.3	38.3	38.3	38.3	38.3
SH	6.6	9.7	12.8	14.3	14.5	14.5	14.5	14.5	14.5	14.5	14.5	15.1	22.0	28.7	32.5	33.6	33.6	33.6	33.6	33.6	33.6
HS	6.6	10.1	15.9	23.1	30.9	36.9	38.3	38.3	38.3	38.3	38.3	38.3	38.3	38.3	38.3	38.3	38.3	38.3	38.3	38.3	38.3
HSH	6.6	9.8	15.8	23.7	29.7	36.4	37.9	38.2	38.2	38.2	38.2	38.2	38.2	38.2	38.2	38.2	38.2	38.2	38.2	38.2	38.2
Sn:	6	NRep:	100	Nloci:	5000	Sloci:	20	Ngen:	20	Nprgy:	50	Sel:	0.1	Dom:	1						
S	4.2	4.1	4.1	4.1	4.1	4.1	4.1	4.1	4.1	4.1	4.1	4.1	4.1	4.1	4.1	4.1	4.1	4.1	4.1	4.1	4.1
H	4.3	5.9	6.4	6.6	6.7	6.7	6.7	6.7	6.7	6.7	6.7	6.7	6.7	6.7	6.7	6.7	6.7	6.7	6.7	6.7	6.7
SH	4.3	4.3	4.3	4.4	4.4	4.4	4.4	4.4	4.4	4.4	4.4	6.4	6.3	6.4	6.4	6.4	6.4	6.4	6.4	6.4	6.4
HS	4.2	5.9	6.5	6.6	6.7	6.7	6.7	6.7	6.7	6.7	6.7	6.7	6.7	6.7	6.7	6.7	6.7	6.7	6.7	6.7	6.7
HSH	4.3	5.9	6.4	6.2	6.7	6.7	6.7	6.7	6.7	6.7	6.7	6.7	6.7	6.7	6.7	6.7	6.7	6.7	6.7	6.7	6.7
Sn:	7	NRep:	100	Nloci:	5000	Sloci:	20	Ngen:	20	Nprgy:	50	Sel:	0.2	Dom:	1						
S	16.6	16.1	16.3	16.4	16.4	16.4	16.4	16.4	16.4	16.4	16.4	16.4	16.4	16.4	16.4	16.4	16.4	16.4	16.4	16.4	16.4
H	16.0	30.0	35.3	37.0	38.1	38.3	38.3	38.3	38.3	38.3	38.3	38.3	38.3	38.3	38.3	38.3	38.3	38.3	38.3	38.3	38.3
SH	16.7	15.4	15.5	15.7	15.7	15.7	15.7	15.7	15.7	15.7	15.7	35.0	33.4	34.9	35.0	35.0	35.0	35.0	35.0	35.0	35.0
HS	16.1	29.9	35.4	37.0	38.1	38.3	38.3	38.3	38.3	38.3	38.3	38.3	38.3	38.3	38.3	38.3	38.3	38.3	38.3	38.3	38.3
HSH	16.1	29.0	35.3	33.2	38.1	38.0	38.3	38.2	38.3	38.27	38.3	38.3	38.3	38.3	38.3	38.3	38.3	38.3	38.3	38.3	38.3

* Gen=generation; Sn=genetic scenario number; NRep=number of replication; Nloci=number of loci; Sloci=number of selective loci; Ngen=number of generation; Nprgy=number of progeny; Sel=selection coefficient (*s*); Dom=dominance level (*h*); S=selfing; H=half-sib; SH=selfing+half-sib; HS=half-sib+selfing; HSH=half-sib+selfing+half-sib

Table S2 Parental heterozygosity over 20 generations in simulated breeding programs with five breeding schemes under seven genetic models

Gen	0	1	2	3	4	5	6	7	8	9	10	11	12	13	14	15	16	17	18	19	20
Sn:	1	NRep:	100	Nloci:	5000	Sloci:	20	Ngen:	20	Nprgy:	50	Sel:	0	Dom:	0						
S	1	0.49	0.25	0.12	0.06	0.03	0.02	0.01	0.00	0.00	0.00	0.00	0.00	0.00	0.00	0.00	0.00	0.00	0.00	0.00	0.00
H	1	0.91	0.85	0.89	0.84	0.79	0.74	0.69	0.63	0.58	0.54	0.50	0.46	0.42	0.39	0.36	0.33	0.30	0.28	0.26	0.23
SH	1	0.91	0.77	0.68	0.64	0.61	0.60	0.59	0.59	0.58	0.58	0.58	0.57	0.54	0.53	0.49	0.46	0.43	0.39	0.36	0.34
HS	1	0.91	0.97	0.93	0.88	0.83	0.77	0.71	0.66	0.61	0.56	0.46	0.37	0.32	0.29	0.28	0.27	0.26	0.26	0.26	0.26
HSH	1	0.91	0.85	0.87	0.66	0.72	0.56	0.58	0.46	0.47	0.37	0.38	0.30	0.31	0.24	0.25	0.20	0.20	0.16	0.16	0.13
Sn:	2	NRep:	100	Nloci:	5000	Sloci:	20	Ngen:	20	Nprgy:	50	Sel:	0.1	Dom:	0						
S	1	0.49	0.24	0.12	0.06	0.03	0.02	0.01	0.00	0.00	0.00	0.00	0.00	0.00	0.00	0.00	0.00	0.00	0.00	0.00	0.00
H	1	0.91	0.85	0.89	0.84	0.79	0.74	0.68	0.63	0.58	0.54	0.49	0.45	0.42	0.38	0.35	0.32	0.30	0.28	0.25	0.23
SH	1	0.91	0.77	0.68	0.64	0.61	0.60	0.59	0.59	0.58	0.58	0.58	0.57	0.54	0.52	0.49	0.46	0.42	0.39	0.36	0.33
HS	1	0.91	0.97	0.93	0.88	0.83	0.77	0.71	0.66	0.61	0.56	0.46	0.37	0.32	0.29	0.28	0.27	0.26	0.26	0.26	0.26
HSH	1	0.91	0.85	0.87	0.65	0.72	0.56	0.58	0.45	0.47	0.37	0.38	0.30	0.31	0.24	0.25	0.20	0.20	0.16	0.16	0.13
Sn:	3	NRep:	100	Nloci:	5000	Sloci:	20	Ngen:	20	Nprgy:	50	Sel:	0.2	Dom:	0						
S	1	0.49	0.25	0.12	0.06	0.03	0.02	0.01	0.00	0.00	0.00	0.00	0.00	0.00	0.00	0.00	0.00	0.00	0.00	0.00	0.00
H	1	0.91	0.85	0.89	0.84	0.79	0.74	0.68	0.63	0.58	0.54	0.49	0.45	0.42	0.38	0.35	0.33	0.30	0.28	0.25	0.23
SH	1	0.91	0.78	0.69	0.64	0.61	0.60	0.59	0.59	0.59	0.59	0.59	0.58	0.54	0.53	0.49	0.46	0.43	0.39	0.36	0.33
HS	1	0.91	0.97	0.93	0.88	0.82	0.77	0.71	0.66	0.61	0.56	0.46	0.37	0.32	0.29	0.27	0.27	0.26	0.26	0.26	0.26
HSH	1	0.91	0.85	0.87	0.65	0.72	0.56	0.58	0.46	0.47	0.37	0.38	0.30	0.31	0.24	0.25	0.20	0.20	0.16	0.16	0.13
Sn:	4	NRep:	100	Nloci:	5000	Sloci:	20	Ngen:	20	Nprgy:	50	Sel:	0.1	Dom:	0.5						
S	1	0.49	0.25	0.12	0.06	0.03	0.02	0.01	0.00	0.00	0.00	0.00	0.00	0.00	0.00	0.00	0.00	0.00	0.00	0.00	0.00
H	1	0.91	0.85	0.89	0.84	0.79	0.74	0.68	0.63	0.58	0.54	0.49	0.46	0.42	0.39	0.35	0.33	0.30	0.28	0.25	0.23
SH	1	0.91	0.77	0.68	0.64	0.61	0.60	0.59	0.59	0.58	0.58	0.58	0.57	0.54	0.52	0.49	0.46	0.42	0.39	0.36	0.33
HS	1	0.91	0.97	0.93	0.88	0.82	0.77	0.71	0.66	0.61	0.56	0.46	0.37	0.32	0.29	0.28	0.27	0.26	0.26	0.26	0.26
HSH	1	0.91	0.85	0.87	0.65	0.72	0.56	0.58	0.45	0.47	0.37	0.38	0.30	0.31	0.24	0.25	0.20	0.20	0.16	0.16	0.13
Sn:	5	NRep:	100	Nloci:	5000	Sloci:	20	Ngen:	20	Nprgy:	50	Sel:	0.2	Dom:	0.5						
S	1	0.49	0.24	0.12	0.06	0.03	0.02	0.01	0.00	0.00	0.00	0.00	0.00	0.00	0.00	0.00	0.00	0.00	0.00	0.00	0.00
H	1	0.91	0.85	0.89	0.84	0.79	0.74	0.68	0.63	0.58	0.54	0.49	0.45	0.42	0.38	0.35	0.33	0.30	0.28	0.25	0.23
SH	1	0.91	0.77	0.68	0.63	0.61	0.60	0.59	0.59	0.58	0.58	0.58	0.57	0.54	0.52	0.49	0.46	0.42	0.39	0.36	0.33
HS	1	0.91	0.97	0.93	0.88	0.82	0.77	0.71	0.66	0.61	0.56	0.46	0.37	0.32	0.29	0.28	0.27	0.26	0.26	0.26	0.26
HSH	1	0.91	0.85	0.87	0.65	0.71	0.56	0.58	0.45	0.47	0.37	0.38	0.30	0.31	0.24	0.25	0.20	0.20	0.16	0.16	0.13
Sn:	6	NRep:	100	Nloci:	5000	Sloci:	20	Ngen:	20	Nprgy:	50	Sel:	0.1	Dom:	1						
S	1	0.49	0.25	0.12	0.06	0.03	0.02	0.01	0.00	0.00	0.00	0.00	0.00	0.00	0.00	0.00	0.00	0.00	0.00	0.00	0.00
H	1	0.91	0.85	0.89	0.84	0.79	0.74	0.68	0.63	0.58	0.54	0.50	0.46	0.42	0.39	0.35	0.33	0.30	0.28	0.25	0.23
SH	1	0.91	0.77	0.69	0.64	0.61	0.60	0.59	0.59	0.59	0.58	0.58	0.57	0.54	0.53	0.49	0.46	0.43	0.39	0.36	0.33
HS	1	0.91	0.97	0.93	0.88	0.83	0.77	0.71	0.66	0.61	0.56	0.46	0.37	0.32	0.29	0.28	0.27	0.26	0.26	0.26	0.26
HSH	1	0.91	0.85	0.87	0.66	0.72	0.56	0.58	0.45	0.47	0.37	0.38	0.30	0.31	0.24	0.25	0.20	0.20	0.16	0.16	0.13
Sn:	7	NRep:	100	Nloci:	5000	Sloci:	20	Ngen:	20	Nprgy:	50	Sel:	0.2	Dom:	1						
S	1	0.49	0.25	0.12	0.06	0.03	0.02	0.01	0.00	0.00	0.00	0.00	0.00	0.00	0.00	0.00	0.00	0.00	0.00	0.00	0.00
H	1	0.91	0.85	0.89	0.84	0.79	0.74	0.68	0.63	0.58	0.54	0.49	0.45	0.42	0.39	0.35	0.33	0.30	0.28	0.25	0.23
SH	1	0.91	0.78	0.69	0.64	0.61	0.60	0.59	0.59	0.59	0.58	0.58	0.57	0.54	0.53	0.49	0.46	0.43	0.39	0.36	0.34
HS	1	0.91	0.97	0.93	0.88	0.83	0.77	0.71	0.66	0.61	0.56	0.46	0.37	0.32	0.29	0.28	0.27	0.26	0.26	0.26	0.26
HSH	1	0.91	0.85	0.87	0.66	0.72	0.57	0.58	0.45	0.47	0.37	0.38	0.30	0.31	0.24	0.25	0.20	0.20	0.16	0.16	0.13

* Gen=generation; Sn=genetic scenario number; NRep=number of replication; Nloci=number of loci; Sloci=number of selective loci; Ngen=number of generation; Nprgy=number of progeny; Sel=selection coefficient (*s*); Dom=dominance level (*h*); S=selfing; H=half-sib; SH=selfing+half-sib; HS=half-sib+selfing; HSH=half-sib+selfing+half-sib
